# Novel *EIF2AK4* mutations in histologically proven pulmonary capillary hemangiomatosis and hereditary pulmonary arterial hypertension

**DOI:** 10.1186/s12881-019-0915-7

**Published:** 2019-11-11

**Authors:** Ossama K. Abou Hassan, Wiam Haidar, Mariam Arabi, Hadi Skouri, Fadi Bitar, Georges Nemer, Imad Bou Akl

**Affiliations:** 1 Departments of Internal Medicine, Faculty of Medicine, American University of Beirut, P.O.Box: 11-0236, Beirut, Lebanon; 2 Departments of Pediatrics and Adolescent Medicine, Faculty of Medicine, American University of Beirut, Beirut, Lebanon; 3 Departments of Biochemistry and Molecular Genetics, Faculty of Medicine, American University of Beirut, P.O.Box: 11-0236, Beirut, Lebanon; 4 Program of Genomics and Precision Medicine, College of Health and Life Sciences, Hamad Bin Khalifa University, Doha, Qatar

**Keywords:** Pulmonary hypertension, EIF2AK4, Hemangiomatosis, Exome, Sequencing

## Abstract

**Background:**

Pulmonary hypertension (PH) remains one of the rarest and deadliest diseases. Pulmonary Capillary Hemangiomatosis (PCH) is one of the sub-classes of PH. It was identified using histological and molecular tools and is characterized by the proliferation of capillaries into the alveolar septae. Mutations in the gene encoding the eukaryotic translation initiation factor 2 alpha kinase 4 (EIF2AK4) have recently been linked to this particular subgroup of PH.

**Methods:**

In our effort to unveil the genetic basis of idiopathic and familial cases of PH in Lebanon, we have used whole exome sequencing to document known and/or novel mutations in genes that could explain the underlying phenotype.

**Results:**

We showed bi-allelic mutations in *EIF2AK4* in two non-consanguineous families: a novel non-sense mutation c.1672C > T (p.Q558*) and a previously documented deletion c.560_564drlAAGAA (p.K187Rfs9*). Our histological analysis coupled with the CT-scan results showed that the two patients with the p.Q558* mutation have PH. In contrast, only one of the individuals harboring the p.K187Rfs9* variant has a documented PCH while his older brother remains asymtomatic. Differential analysis of the variants in the genes of the neighboring network of *EIF2AK4* between the two siblings identified a couple of interesting missense mutations that could account for this discrepancy.

**Conclusion:**

These findings represent a novel documentation of the involvement of *EIF2AK4* in the different aspects of pulmonary hypertension. The absence of a molecular mechanism that relates the abrogated function of the protein to the phenotype is still a major hurdle in our understanding of the disease.

## Background

Pulmonary capillary hemangiomatosis (PCH) is a rare but increasingly recognized cause of pulmonary hypertension [1]. Although it was first described in 1978, fewer than 50 cases have been reported in the literature since [2]. The median survival is 3 years and the only effective therapy is lung transplantation. It is now classified in the diagnostic group 1′, a distinct subgroup of pulmonary arterial hypertension. PCH is a diffuse process characterized by the proliferation of capillaries into the alveolar septae, often invading small vessels and bronchi [3]. It is combined in classification with pulmonary veno-occlusive disease (PVOD) [4]. Indeed, PCH and PVOD overlap in several histopathological findings such as venous obstruction, arterial intimal fibrosis and interlobular septal thickening. They also share clinical, radiological, and hemodynamic characteristics [5, 6].

Patients with PCH present with nonspecific symptoms of dyspnea, fatigue, cough, and hemoptysis. Echocardiogram and right heart catheterisation show changes compatible with pulmonary arterial hypertension (PAH) and similar to those found in other forms of PAH. High resolution CT scans of the chest show centrilobular ground glass opacities, septal lines, and mediastinal lymph node enlargement, findings common to PVOD [2]. Despite clinical suspicion of PCH, a definitive diagnosis requires histological examination of lung tissue and is often delayed until lung transplant and the examination of explanted tissue. Treatment of PCH with PAH-specific therapy has been described as a bridge to lung transplant [7]. Response to PAH-specific therapy is limited. Drug initiation and titrations can induce pulmonary edema and should be performed with caution [2, 7–9].

Sporadic and familial cases of PCH have been reported, indicating a genetic component in the etiology of the disease. Mutations in the *EIF2AK4* gene (coding for eukaryotic translation initiation factor 2α Kinase 4) were identified as the genetic predisposition causes of PCH. These mutations were first found in an autosomal recessively inherited PCH familial case and in 20% of sporadic cases [10]. *EIF2AK4* mutations are also found in PVOD patients, reinforcing the link of these two diseases to a common genetic risk factor [11–13].

We report a new *EIF2AK4* mutation type in a histologically proven case of PCH. It is also the first reported case of *EIF2AK4* mutation in PCH in the Eastern Mediterranean region (Lebanese population). We also report a novel *EIF2AK4* homozygous mutation in a family clinically diagnosed with HPAH.

## Methods

### Subjects and clinical characteristics

Patients and their family members when available were recruited as part of a clincical and genetic research study on PAH in Lebanon between 2015 and 2017. They were eligible for enrolment if the patients had a pulmonary arterial pressure (mPAP) > 25 mmHg at rest and a pulmonary artery wedge pressure (PAWP) < 15 mmHg.

The study was approved by the Institutional Review Board (IRB) at the American University of Beirut Medical Center (AUBMC) (Protocol Number IM.IB.01). Written consent forms were collected from all participants. The collected data include medical history, physical exam, family history for PAH, echocardiography, pulmonary function test, chest computerized tomography (CT), CT pulmonary angiography, ventilation perfusion scan, and pro-Brain Natriuritic Peptide (BNP) blood levels.

### Genetic studies

Five millileters of peripheral blood were collected from the patients. We used the Qiagen QIAamp blood midi-kit to extract DNA and we assessed its purity using Nanodrop (Thermo Fisher) at the molecular core facility at AUB. Library preparation and subsequent exome capture was done on an Illumina HiSeq2500 platform at Macrogen (South Korea) using the V5 SureSelect kits (Agilent). Sequences were aligned to the hg19 human genome using Novoalign and variants were called by the Genome Analysis Toolkit (GATK). We used the variant call software (Illumina) to further annotate and analyze the obtained variants. Sanger sequencing was carried on with specific primers to confirm the mutations in the affected and no-affected individuls using the ABI3500 platform at AUB.

### Variant analysis

Analysis was performed based on a filtering panel consisting of genes (Additional file 4: Table S1) implicated in PAH as previously described [14]. Only non-synonymous, insertion/deletions variants in the coding regions, and splicing variants with allele frequencies inferior to 1% were filtered in according to their evolutionary conservation, and location within the encoded protein. These criteria were evlauted by in silico predictive software (SIFT: https://sift.bii.a-star.edu.sg/ and Polyphen2: http://genetics.bwh.harvard.edu/pph2/) and scored accordingly (Additional file 1: Figure S1).

## Results

### A sporadic case of PCH with a previously documented *EIF2AK4* variant

**Fig. 1:**
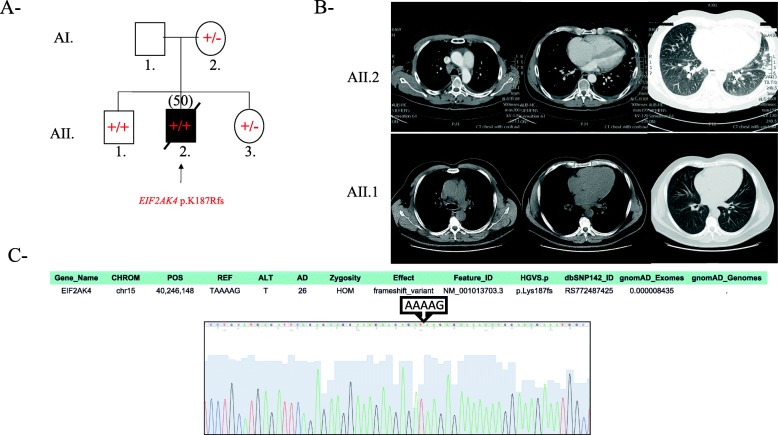
Clinical and genetic characterization of Family A patients. **a** Pedigree for the family, showing the frameshift stop-gained EIF2AK4 mutation. The −/− symbol is for normal genes, −/+ is for heterozygous mutations and the +/+ is for homozygous mutations. Male; Circle, female; open symbol, unaffected; filled symbol, affected, the symbol with arrow: Death. Current age or age at death in years is between parenthesis. **b** The chest CT scan of the index patient (AII.2) with a clinical presentation of Pulmonary veno-occlusive disease shows dilated pulmonary trunk, small pleural, pericardial effsuions, and interlobular septal thickening in the lung bases. The lower panel is the asymptomatic older brother’s (AII.1) CT scan which doesn’t show any abnormality in the chest. **c** Sanger chromatogram tracing confirming the NGS variant sequence for both AII.1 and AII.2, and showing the *EIF2AK4* mutation as detailed in the supp. Table from whole exome sequencing results

**Fig. 2:**
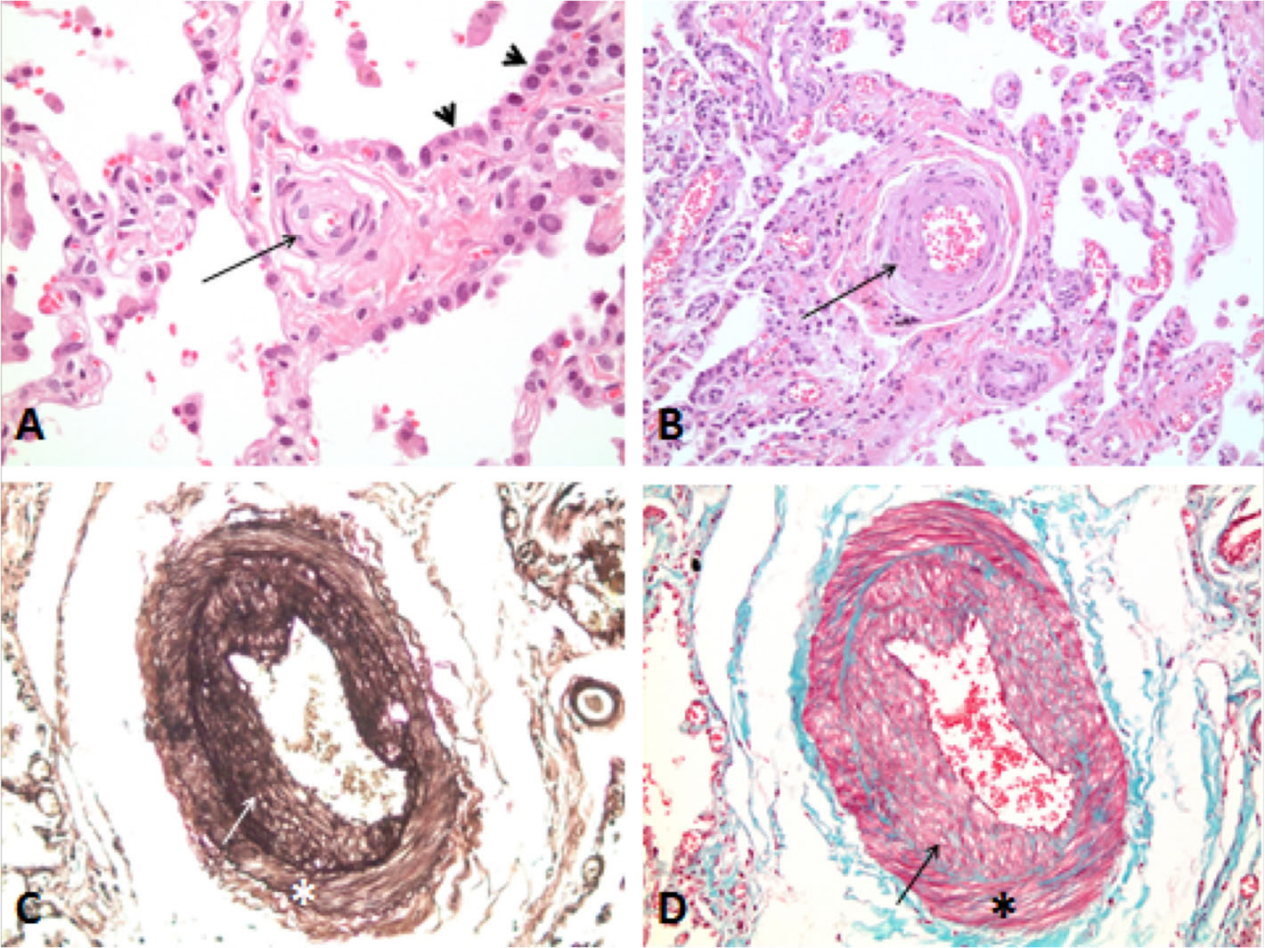
Histopathologic findings on open lung biopsy of patient AII.2. The alveolar septa are highly thickened (**a**). Small and intermediate size veins show thickened walls and narrowed lumens due to concentric laminar fibrosis (**a** and **b** respectively). Medium and large arteries have a thickened media and prominent intimal fibrosis (**c**,**d**). Symbols: *, intima; arrow, media; arrow head, alveolar septa

The indexed case is a 47-year-old man, non-smoker, who presented to the hospital for progressive dyspnea on exertion of 1 year duration (Fig. 1a). The dyspnea’s onset was insidious, progressed rapidly and affected his daily activity. He reported mild lower extremity edema, but no orthopnea, no paroxysmal nocturnal dyspnea (PND), no wheezing, and no cough. CT angiography of the chest showed no pulmonary embolism nor thickening of interlobular septae (Fig. 1b). The transthoracic echocardiogram showed normal left ventricular function with a moderately dilated right ventricle and a pulmonary arterial systolic pressure of 75 mmHg. Spirometry was also performed and showed no flow obstruction and normal FVC (Functional Vital Capacity). Cardiac MRI showed no evidence of shunt or infiltrative cardiomyopathy. Right heart catheterization showed severe Pulmonary Artery Hypertension (PAH) with a mean pulmonary artery pressure (mPAP) of 68 mmHg and pulmonary wedge pressure of 13 mmHg. The pulmonary vascular resistance was calculated to be 13 Wu. The 6-min-walk was 368 m. An open lung biopsy was performed and it showed that the alveolar septae were thickened with prominent intimal fibrosis and medial thickening of the veins (Fig. 2). Apart from the presence of rare interstitial lymphocytes and mildly increased alveolar macrophages, there was no significant inflammation. These findings confirmed the suspicion of PCH. The patient was started on Sildenafil and the dose was slowly titrated to 25 mg TID. He experienced only mild improvement, which prompted the addition of Bosentan to alleviate the symptoms. He continued to deteriorate over the next 2 years leading to his death 3 years after the initial diagnosis.

Whole exome sequencing was perfomed on the patient, and after filtering in variants with a minor allele frequency (MAF) < 1%, a bi-allelic mutation in *EIF2AK4* was detected. The p.K187 fs mutation (rs772487425) was previously reported in a 32-year-old female with a documented histological PVOD phenotype [12, 15]. In our case, the patient had no family history of pulmonary hypertension and had two siblings. Sanger sequencing confirmed that the patient (AII.2) had the homozygous mutation along with his older brother (AII.1) (Fig. 1c). His sister (AII.3) and mother (AI.2) were heterozygous for the mutation. The father (AI.1) did not consent to participate in the study. Clinical workout of AII.1 showed, at 50 years of age, no echocardiographic findings suggestive of pulmonary arterial hypertension. A CT scan of the chest was normal with no centrilobular ground glass opacities, no septal lines, and no mediastinal lymph node enlargement which would have suggested that he had PCH or PVOD like his brother (Fig. 1b). His pulmonary function test was normal. This prompted us to further cross-compare the results of the exome sequencing between the two siblings with bi-allelic *EIF2AK4* mutation in order to account for the discrepancy in the phenotype. We based our hypothesis on a potential variant that would have “a gain of function” effect on the neighbouring network of genes around *EIF2AK4* (Additional file 2: Figure S2). None of the other EIF gene encoding kinases displayed a differential variant signature between AII.1 and AII.2 (Additional file 5: Table S2). Interestingly, only one missense variant p.V682 M in the gene encoding GCN1L1 (general control of amino acid synthesis-1 like protein 1), an *EIF2AK4* obligate partner was detected in the unaffected individual with a minor allele frequency of 0 (Additional file 6: Table S3).

### A familial case of pulmonary hypertension with a novel mutation in *EIF2AK4*

**Fig. 3:**
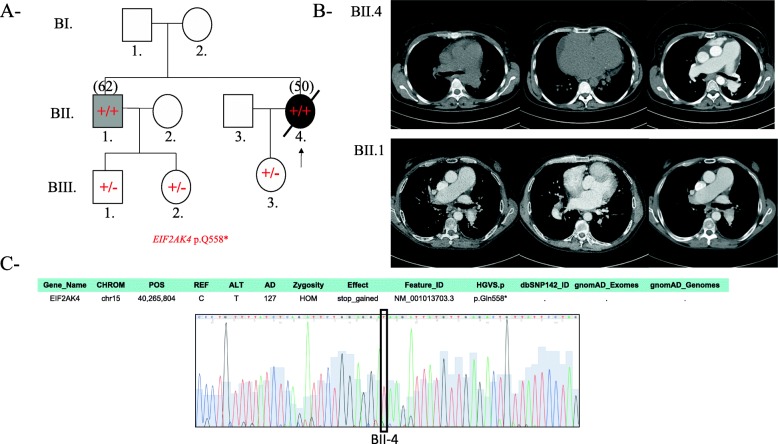
Clinical and genetic characterization of Family A patients. **a** Pedigree for the family, showing the frameshift stop-gained *EIF2AK4* mutation. The −/− symbol is for normal genes, −/+ is for heterozygous mutations and the +/+ is for homozygous mutations. Male; Circle, female; open symbol, unaffected; filled symbol, affected, the symbol with arrow: Death. Current age or age at death in years is between parenthesis. **b** The chest CT scan of the index patient (BII.4) with a clinical presentation of hereditary pulmonary arterial hypertension shows normal parenchyma and minimal pericardial effusion. The lower panel is the older brother’s (BII.1) CT scan which shows enlarged pulmonary artery and no parenchymal changes. Both Images did not have neither pleural effusion, nor centrilobular nodules. **c** Sanger chromatogram tracing confirming the NGS variant sequence for the patient and showing the mutation as detailed in the supp. Table from whole exome sequencing results

The indexed patient BII.4 is a woman who presented at the age of 36 years with slowly progressing dyspnea on exertion of 1 year duration (Fig. 3a). Her diagnostic work up started with a CT angiography that showed a dilated pulmonary artery suggestive of PAH, normal parenchyma, and no pulmonary embolism. Transthoracic echocardiogram showed normal left ventricular function with tricuspid regurgitation and an estimated pulmonary artery systolic pressure of 90 mmHg. The mPAP by right heart catheter was 45 mmHg. She was diagnosed with idiopathic PAH and started on Sildenafil with good response. She remained stable for 7 years, at which time Bosentan was added. She remainded stable for another 7 years, at which time she developed severe community acquired pneumonia and ARDS and passed away at the age of 50 years. 6 years following her initial diagnosis, her brother (BII.1) presented with similar symptoms at the age of 51, while the parents did not participate in the study. The brother’s mPAP was 50 mmHg and he was started on a Sildenafil-Bosentan combination therapy and remained stable 8 years later with good functional status. Like his sister, patient BII.1 had good response to PAH targeted therapy with no episodes of congestion upon the initiation of the vasodilators. Once we found the novel homozygous nonsense – p.Q558*- mutation in *E1F2AK4* in both patients (Fig. 3c), high resolution chest CTs were repeated and reviewed. Both patients had no radiological changes suggestive of PVOD or PCH (Fig. 3b).

## Discussion

This is the first documentation of *EIF2AK4* variants in Lebanon, a small country in the Eastern Mediterranean region with consanguineous marriage rates that remain relatively high. On one hand, we have a patient with a previously reported *EIF2AK4* mutation presenting as a sporadic PCH case confirmed by histological findings and radiological profile while his two-years older brother who carries the same mutation has no signs or symptoms of the disease. On the other, we describe for the first time a family presenting with a novel homozygous *EIF2AK4* mutation with a confirmed clinical picture of hereditary PAH.

PCH/PVOD is a rare cause of precapillary pulmonary hypertension and can mistakenly be diagnosed as idiopathic PAH. Diagnosis of PCH is based on clinical suspicion. High resolution CT scan findings of centrilobular ground glass opacities, subpleural thickened septal lines, and mediastinal lymphadenopathy, can provide clues for the diagnosis [1, 2]. Prevalence of biallelic EIF2AK4 mutation is 9% in the sporadic group of patients and may help, when present, to confirm PCH/PVOD diagnosis by performing genetic testing and avoiding a more risky confirmation by biopsy [2, 5]. Our first patient presented at age 46 and had advanced New York Heart Association (NYHA) functional class on presentation which is common among patients with PCH/PVOD. The diagnosis of PCH was confirmed by histologic examination of an open lung biopsy. He had mild improvement of his symptoms with PAH-targeted therapy of PDE5 inhibitor and Endothelin Receptor Antagonist (ERA) along with diuretics. He did not develop pulmonary edema after the initiation of therapy which can happen in PCH/PVOD patients [2].

The p.K187Rfs mutation in *EIF2AK4* detected in our second patient was previously reported in a patient diagnosed at the age of 32 with PCH/PVOD [12, 15]. Our patient was much older at the onset of diagnosis and his older brother (now 51) remains unaffected. Our patient, and his unaffected brother, had no exposure to solvent or chemotherapy, which are reported causes of PCH/PVOD [9, 16]. These results suggest that the “penetrance” of the mutations in *EIF2AK4* is similar to other familial cases of PAH whereby mutations in *BMPR2* are partially penetrant [17–19], and/or that environmental factors including diet could be implicated in the differential phenotype observed so far [8, 15]. Previous studies have argued that mutations in *EIF2AK4* lead either to a defective protein or to the absence of expression of the protein. However, there are no functional studies so far to assess the effect of missense mutations or truncated protein products on the activity of the encoded EIF2AK4 protein despite a recent attempt to quantify the protein’s expression in postmortem lung tissues [20]. It is even perplexing that the *Eif2ak4*^*−/−*^ mouse model did not show any substantial phenotype pertaining to PCH/PVOD [21]. The explanation could be related to a genetic/genomic background “elevated potential “that compensates for the abrogated activity of the mutated protein.

It is clear that little is known with respect to the *EIF2AK4* mutation’s penetrance due to the rare disease and difficult diagnosis. There are suggestions to the fact that even heterozygous compound mutations might manifest as PH supporting a “second hit” phenomenon [22]. We have selected to overview some of the genes in the close vicinity of the *EIF2AK4* network (Additional file 2: Figure S2). There are three additional enzymes that could phosphorylate the elongation initiation factor type 2 (EIF2a) encoded by the genes *EIF2AK1–3*; none showed differential variants between the two siblings (Additional file 5: Table S2). On the contrary the EIF2AK4 activating cofactor GCN1L1 was shown to harbor a missense variation only in the phenotypically normal individual. This variant is unique since it is not found in the GNOmad exomes and genomes and is predicted to have a mild effect on protein function. We hypothesize that this variant would confer a gain of function activity that enhances its interaction with EIF2AK4, although the domain of interaction with the latter is situated in the C-terminal region, which is deleted in the presumably truncated protein [23, 24]. Alternatively, a different diet regimen may be responsible for the “phenotypic” difference between the siblings, taking into consideration that EIF2AK4 has been shown to prevent oxiditative damage caused by an amino-acid unbalanced diet [25, 26]. Finally, additional variants in genes with no documented implication in PCH/PVOD could also account for this discrepancy. Clinical follow up is needed for the individual at risk as the phenotype expression could appear later, as was the case in the founder *EIF2AK4* mutation in the Iberian Romani population [11].

## Conclusion

Despite the breakthrough in the identification of EIF2AK4 as a major player in PVOD/PCH, little is known of its implication in PAH, and much of the controversy linked to this issue is related to the proper radiological and clinical evidence that supports or refutes the causal relationship. The first report of a mutation in *EIF2AK4* reponsible for hereditary pulmonary arterial hypertension came as a combined heterozygous mutation in conjunction with a *BMPR2* mutation [22]. Since then, few large cohort studies have shown hereditary pulmonary arterial hypertension bi-allelic mutations in *EIF2AK4*. These mutations were also described in 1% of patients presenting with a clinical diagnosis of idiopathic PAH. The patients had a worse prognosis when compared with other PAH patients within the same cohort. They tended to have more radiological findings that are found in PCH/PVOD than PAH patients without the mutation. This was also the case in the BRIDGE study whereby 9 patients out of 864 with either idiopathic PAH or hereditary PAH had bi-allelic *EIF2AK4* mutations [5]. Our patients with the novel homozygous *EIF2AK4* mutation p.Q558* were clinically diagnosed with hereditary PAH, and had no findings suggestive of PCH/PVOD. The presence of the biallelic mutation was the first clue to a possible PCH diagnosis. Unlike patients with PCH/PVOD, they both had good response to vasoldilators therapy with improvement in their functional status. In contrast to the rapidly progressive nature of PCH/PVOD, the disease seemed to remain stable for an extended period of time. The chest CT of our patients did not show interlobular septal thickening as is the case in previous studies of PAH patients with bi-allelic mutations in *EIF2AK4* [5, 10, 27]. These genetic, clinical, and radiological findings raise the question about the functional properties of the mutated EIF2AK4 protein products and whether specific domains of the protein are implicated in divergent signaling pathways. Indeed, our compilation of the described mutations in *EIF2AK4* causing PCH/PVOD or PAH show a clustering of mutations in the His-tRNA synthase like domain of the protein for patients with PCH/PVOD (Additional file 3: Figure S3). Structure-function studies are warranted to delineate the phenotype/genotype correlations and better understand the molecular pathways involved in pulmonary hypertension.

## Supplementary information

**Additional file 1: Figure S1.** Variant filtering workflow for exome sequencing of the patients. The left panel illustrates the type of unselected variants in the filter followed by exclusion of non-disease causing identified in polyphen, the right panel show the selected variants included in the filter followed by including all the allele frequencies < 10% to reach 50–70 identified causal variants associated with the disease. UTR = Untranslated Region; AF = allele frequency.

**Additional file 2: Figure S2.** Predicted functional partners of the EIF2AK4 protein. https://string-db.org/cgi/network.pl?taskId=J7XBBrbsBLAi

**Additional file 3: Figure S3.** Current and prior reported *EIF2AK4* mutations detected in extensively worked up patients with PAH, PCH and PVOD diagnosis [5, 11, 12, 27]. The locations of the mutations are depicted on the rightmost and leftmost column and the consequence of the homozygous (blue) or compound mutations (red) and are defined as follows: The vertical black line depicts the protein structure for each patient reported, a point denotes a SNP mutation or stop codon differentiated by the COOH terminal continuation of the protein line structure, a solid red or blue line depicts a deletion, a diagonal line depicts a splice mutation, a dashed line denotes the affected/deleted haplo-insufficiency in relation to the compound chromosome mutation. The functional domains of the protein with respect to the mutation location is depicted in the middle

**Additional file 4: Table S1.** List of Genes Linked to Pulmonary Arterial Hypertension.

**Additional file 5: Table S2.** Variants in the coding regions of the *EIF2AK1–4* genes in Individulas AII1 and AII.2 (excluding synonymous variants).

**Additional file 6: Table S3.** Variants in the coding regions of the *EIF2AK4* gene network in Individulas AII1 and AII.2 (excluding synonymous variants).

## Data Availability

The datasets used and/or analyzed during the current study are available from the corresponding author on reasonable request. Exome sequencing files are available to share through a direct request process to the corresponding authors.

## Abbreviations

ACE: Angiotensin converting enzyme inhibitors
ARDS: Acute respiratory distress syndrome
AUB: American University of Beirut
AUBMC: American University of Beirut Medical Center
BMPR2: Bone morphogenetic protein receptor type 2
BNP: Brain Natriuretic Peptide
BNP: Pro-B-type natriuretic peptide
CT: Computerized tomography
EIF: Eukaryotic initiation factor
*EIF2a*: Elongation initiation factor type 2
EIF2AK1–3: Eukaryotic translation initiation factor 2 alpha kinase 1–3
EIF2AK4: Eukaryotic translation initiation factor 2 alpha kinase 4
ERA: Endothelin Receptor Antagonist
GATK: Genome Analysis Toolkit
GCN1L1: General control of amino acid synthesis-1 like protein 1
gnomAD: Genome Aggregation Database
HPAH: Heritable pulmonary arterial hypertension
IPAH: Idiopathic pulmonary arterial hypertension
IRB: Institutional review board
MAF: Minor allele frequency
mPAP: Mean pulmonary arterial pressure
MRI: Magnetic resonance imaging
PAH: Pulmonary arterial hypertension
PAP: Pulmonary arterial pressure
PAWP: Pulmonary artery wedge pressure
PCH: Pulmonary capillary hemangiomatosis
PDE5: Phosphodiesterase type 5
PDGFs: Platelet-derived growth factors
PH: Pulmonary hypertension
PolyPhen-2: Polymorphism Phenotyping v2
PVOD: Pulmonary veno-occlusive disease
SIFT: Sorting Intolerant from Tolerant
SNP: Single Nucleotide Polymorphism
TID: Three times a day
VEGFs: Vascular endothelial growth factors
WHO: World Health Organization’s
WU: Wood Unit
