# Supplementary material for: Novel EIF2AK4 mutations in histologically proven pulmonary capillary hemangiomatosis and hereditary pulmonary arterial hypertension
Source: BMC Med Genet. 2019 Nov 11;20:176. doi: 10.1186/s12881-019-0915-7 (PMC6849225; doi:10.1186/s12881-019-0915-7)
Supplement: Supplementary file 1 — Additional file 1: Figure S1. Variant filtering workflow for exome sequencing of the patients. The left panel illustrates the type of unselected variants in the filter followed by exclusion of non-disease causing identified in polyphen, the right panel show the selected variants included in the filter followed by including all the allele frequencies < 10% to reach 50–70 identified causal variants associated with the disease. UTR = Untranslated Region; AF = allele frequency. [file 12881_2019_915_MOESM1_ESM.pdf]

Supplementary Figure 1

Exclusion filters

- 3’and 5’ UTR variant,
- downstream gene variant,
- intergenic region,
- Intronic region,
- no-coding exon variant,
- splice acceptor and donor variant,
- splice region variant,
- synonymous variant
- upstream gene variant

- Not Identified by at least two of POLYPHEN, POLYPHEN2 and PROVEAN as disease causing

78K-97K detected variants

50- 70 filtered variants

Inclusion filters

- Single nucleotide variants in the coding regions:
  - a- Missense
  - b- Stop codon gained and lost
- Inframe deletions and insertions
- Frameshift deletions and Insertions

- Allele frequency (1000GP3_AF) to < 1%

Patient	# of SNPs	Missense Var	Stop gained	Stop lost	Frameshift Var	Het/Hom Ratio
13.1	78,595	10,294	90	38	271	1.3
13.2	96,911	11,274	110	46	333	1.5
14.1	81,277	10,549	94	34	275	1.4
14.2	82,234	10,626	94	35	290	1.5
