# Supplementary material for: Novel EIF2AK4 mutations in histologically proven pulmonary capillary hemangiomatosis and hereditary pulmonary arterial hypertension
Source: BMC Med Genet. 2019 Nov 11;20:176. doi: 10.1186/s12881-019-0915-7 (PMC6849225; doi:10.1186/s12881-019-0915-7)

Supplementary Figure 2

EIF2AK4	*Eukaryotic translation initiation factor 2 alpha kinase 4; Metabolic-stress sensing protein kinase that phosphorylates the alpha subunit of eukaryotic translation initiation factor 2 (eIF-2-alpha/EIF2S1) on 'Ser-52' in response to low amino acid availability (PubMed-25329545). Plays a role as an activator of the integrated stress response (ISR) required for adapatation to amino acid starvation. Converts phosphorylated eIF- 2-alpha/EIF2S1 either to a competitive inhibitor of the translation initiation factor eIF-2B, leading to a global protein synthesis repression, and thus to a reduced [...] (1649 aa)*

Predicted Functional Partners:

Protein	Description	Neighborhood	Gene Fusion	Cooccurence	Coexpression	Experiments	Databases	Textmining	[Homology]	Score
EIF2S1	*Eukaryotic translation initiation factor 2, subunit 1 alpha, 35kDa; Functions in the early steps of protein synthesis by forming a ...*									0.999
EIF2S2	*Eukaryotic translation initiation factor 2, subunit 2 beta, 38kDa; eIF-2 functions in the early steps of protein synthesis by formi...*									0.991
GCN1L1	*GCN1 general control of amino-acid synthesis 1-like 1 (yeast); Acts as a positive activator of the EIF2AK4/GCN2 protein kinas...*									0.970
EIF2S3	*Eukaryotic translation initiation factor 2, subunit 3 gamma, 52kDa; As a subunit of eukaryotic initiation factor 2 (eIF2), involved...*									0.952
DNAJC3	*DnaJ (Hsp40) homolog, subfamily C, member 3; Involved in the unfolded protein response (UPR) during endoplasmic reticulu...*									0.899
PPP1CC	*Protein phosphatase 1, catalytic subunit, gamma isozyme; Protein phosphatase that associates with over 200 regulatory prot...*									0.872
FARS2	*phenylalanyl-tRNA synthetase 2, mitochondrial; Is responsible for the charging of tRNA(Phe) with phenylalanine in mitochondr...*									0.871
ATF4	*Activating transcription factor 4 (tax-responsive enhancer element B67); Transcriptional activator. Binds the cAMP response e...*									0.849
PPP1CA	*Protein phosphatase 1, catalytic subunit, alpha isozyme; Protein phosphatase that associates with over 200 regulatory protein...*									0.835
EIF2B5	*Eukaryotic translation initiation factor 2B, subunit 5 epsilon, 82kDa; Catalyzes the exchange of eukaryotic initiation factor 2-bo...*									0.831

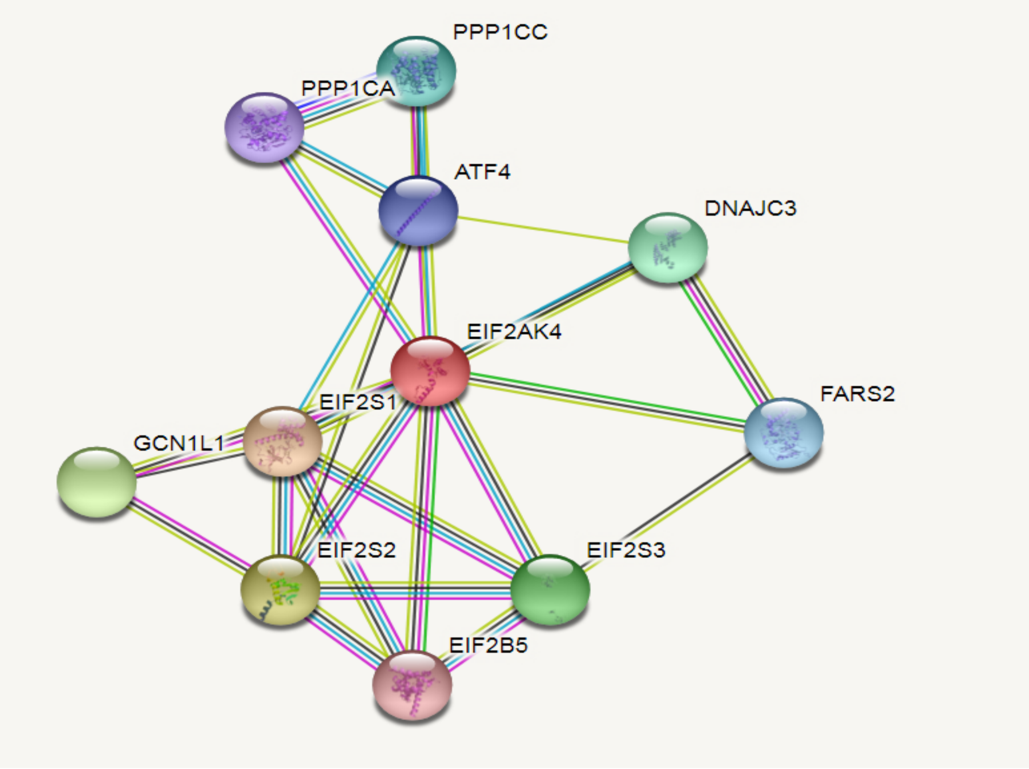

Supplement: Supplementary file 2 — Additional file 2: Figure S2. Predicted functional partners of the EIF2AK4 protein. https://string-db.org/cgi/network.pl?taskId=J7XBBrbsBLAi [file 12881_2019_915_MOESM2_ESM.pdf]
