# Supplementary figures and images for: Novel EIF2AK4 mutations in histologically proven pulmonary capillary hemangiomatosis and hereditary pulmonary arterial hypertension
Source: BMC Med Genet. 2019 Nov 11;20:176. doi: 10.1186/s12881-019-0915-7 (PMC6849225; doi:10.1186/s12881-019-0915-7)

# Supplementary Figure 3

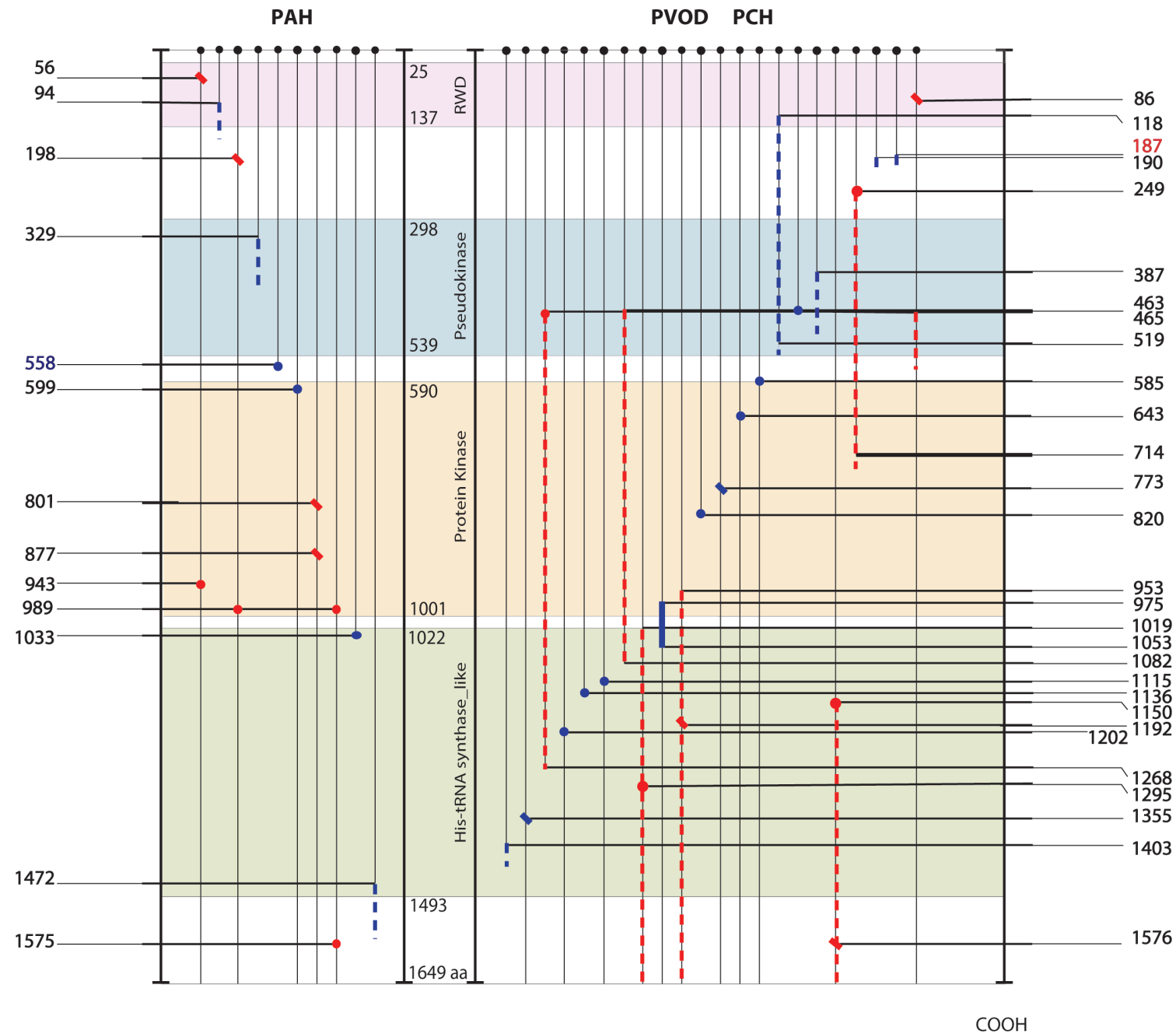

Supplement: Supplementary file 3 — Additional file 3: Figure S3. Current and prior reported EIF2AK4 mutations detected in extensively worked up patients with PAH, PCH and PVOD diagnosis [5, 11, 12, 27]. The locations of the mutations are depicted on the rightmost and leftmost column and the consequence of the homozygous (blue) or compound mutations (red) and are defined as follows: The vertical black line depicts the protein structure for each patient reported, a point denotes a SNP mutation or stop codon differentiated by the COOH terminal continuation of the protein line structure, a solid red or blue line depicts a deletion, a diagonal line depicts a splice mutation, a dashed line denotes the affected/deleted haplo-insufficiency in relation to the compound chromosome mutation. The functional domains of the protein with respect to the mutation location is depicted in the middle [file 12881_2019_915_MOESM3_ESM.pdf]
