# Supplementary material for: Novel EIF2AK4 mutations in histologically proven pulmonary capillary hemangiomatosis and hereditary pulmonary arterial hypertension
Source: BMC Med Genet. 2019 Nov 11;20:176. doi: 10.1186/s12881-019-0915-7 (PMC6849225; doi:10.1186/s12881-019-0915-7)
Supplement: Supplementary file 4 — Additional file 4: Table S1. List of Genes Linked to Pulmonary Arterial Hypertension. [file 12881_2019_915_MOESM4_ESM.pdf]

Supplementary Table 1: List of Genes Linked to Pulmonary Arterial Hypertension					
PDK4	PLOD1	TGFBR1	CTGF	APOE	ACVRL1
THSD7A	HSD17B2	ENG	PKD2	ACE2	ESR2
PDK2	NOX4	CXCL12	SPP1	GDF15	FBLN5
CCL3	HSD17B14	BMPR1A	ESRRB	COL5A1	IGF1R
TAC1	ADAMTS2	ACTA2	KCNIP2	GATA5	NOL3
TBXA2R	MMP2	SLC6A4	CRHR1	HSD17B3	GATA6
NOS2	DNM1L	CCL2	DUSP1	ANO1	TP53
CELSR3|NCKIPSD|SLC26A6	*GP6*	HSD17B1	TEK	PDHA1	SMAD4
SS18L2	FKBP1A	PPARGC1A	TNFSF11	GCH1	FBXO15
ABHD5	F11	CCND1	SMAD9	DNAJB1	CBLN2
ALOX5	PEBP1	VWF	TGFBI	PPARG	AKT1
HGF	TBX5	TRPV4	NPPB	HSD17B7	RHOB
CPS1	NOS1	CDKN1B	TBX2	ERAL1	MYH15
FHL1	PCBP4	SOD2	TBX4	CRP	AGTR1
HSD17B6	ICAM1	VEGFA	CCRL2	CASP9	TCTA
CD44	APOH	HMGCR	TNFSF10	TRPC4	SRD5A1
CASR	ESR1	SMAD5	LRP1	CNDP2	DYNLT1
TNC	MAPK1	PDGFRB	IL13RA2	HSD17B4	VIP
HSPA5	XBP1	HRG	EDN3	PTPN22	EGFR
MAP4	HMOX1	WNT5A	PTGIS	ROCK2	EBAG9
UTS2	PDGFB	GNAT1	PGIS	APLNR	NOTCH1

ELN	HIF1A	VIPR1	PMEPA1	APLNR	HSD17B12
CYBA	MMP9	IL1A	WNT1	PDGFRA	MMP3
COL11A1	NFATC2	ACVR1	BMP4	HTR1B	KLHL1
GUCY1B3	ANGPT4	IL18BP|PDE1A	C3	OS9	CD226
CASP8	SAMHD1	PDE1A	BMP2	HTR2B	CNDP1
MYLK	SMAD7	IFIH1	GDF5	RNASEH2B	EDNRA
RHOA	XIAP	RTN4	ID1	EDNRB	PDK1
ROCK1	CD40LG	FN1	CAPNS1	IL6	GUCY1A2
PDK3	TIMP1	STAT1	CCR7	BLK	BANK1
TGFBR3	KLF5	IL1R1	EDN2	GATA4	ANGPT1
PRKACA	MLYCD	PROC	KDR	PIM1	PRKCA
HSD17B10	FOXF1	ID2	ADM2	IRF4	PDE1C
NFATC3	ATP8B4	ANGPTL1	ADORA2A	TRPC6	SMAD8
PTGS2	PDGFRL	HDAC1	ATF4	THBS1	FBXO32
NOTCH3	RNASEH2A	LEPR	IRF5	CYP19A1	ERG
TIMM21	FCER2	LEPR|LEPROT	GDF2	BCAR3	XDH
FOSL2	TGFB1	CTH	EIF2AK4	CYP1B1	HK2
NFKB2	CAV1	ECE1	EIF2AK4	STAT4	ACE
EDN1	VIPR2	TNFSF4	CCRL1	BMPR1B	PTGIR
SENP1	FKBP14	MUC5B	TPH1	PRDM5	CBS
CPB2	SERPINE1	APOA1	SHBG	TRPC3	ADAR1
ARG2	MEOX2	ATF6	KCNA5	EGF	TNFSF12|TNFSF13

VANGL1	LIMK1	TNFAIP3	RPL29	FBN2	UCN
VCAM1	DAG1	MIRLET7A3	LOC100301990	SMAD3	ATP6V0A2
BMP10	MST1	MIR21	PSC	CTNNB1	PRKG1
TGFBR2	LEP	MIRLET7C	SCAANT1	CX3CR1	ZNF197
TopBP1	SMAD2	MIR210	SLSN3	HLA-DPB1	HEXIM1
RYK	AMIGO3	MIRLET7F1	SLI3	COL3A1	FAM69C
CCR1	MTHFR	MIR30C2	TDGF1P6	STAT3	MAOA
PBRM1	ARIH2	MIRLET7D	DFNA18	CHST14	HLA-DRB1
CDC25A	TSHZ1	MIRLET7G	CACNA1C	IL13	S100A4
ZNF589	ZADH2	MIRLET7A1	FAUP1	CXCL10	ESRRG
DUSP7	NKX2-6	BMPR2	BRCA3	MAP3K2	MMP1
GUCY1A3	TH	HSD17B8	DHX9P1	FOS	CD47
F2RL1	F2	COL5A2	EEF1A1P3	SMAD1	ARHGEF12
SHROOM1	PRF1	TNF	ATXN8	HSD17B13	SRC
COL1A2	UTS2R	POU5F1	F5	HAS2	SERPINA1
GPER1	TMIE	POU5F1|POU5F1P1	FRA13B	APLN	PDGFA
NOS3	UBA7	GNB2L1	HNRNPA1P11	LAMB2	SLC2A10
CFL2	NKX2-5	C18orf63	KCNK3	NME6	HES5
SLC39A13	SCN5A	HLA-DQB1	HSCRS2	PRL	PCDH20
FBN1	PROS1	MIR224	PPP2R3A	CXCR6	HSD17B11
GREM1	TMEM173	MIR27A	NCK1	CEBPB	HLA-DRB5
ATXN8OS	LTBP1	MIR27B	MRAS	CFL1	RYR2

PSMD10P3	RASGRP3	MIRLET7B	PIK3CB	RNASEH2C	SMURF1
LOC100529111	PIBF1	MIR204	SPSB4	ESRRA	MIRLET7E
RPL35AP8	KCTD12	MIR328	RASA2	MIR17	PLB1
LOC400655	EDNRB	MIR503	ATP1B3	LOC100505876	PPP1CB
LOC100132647	SPRY2	MIR130A	GBE1	MIR424	LBH
MIR451A	COL2A1	MIRLET7F2	RAB19	STGC3	CAPN13
LOC400657	CELIAC9	NCKIPSD	GPR113	ZNF469	NLRC4
MIR145	MIRLET7A2	TREX1			
