# Supplementary material for: Novel EIF2AK4 mutations in histologically proven pulmonary capillary hemangiomatosis and hereditary pulmonary arterial hypertension
Source: BMC Med Genet. 2019 Nov 11;20:176. doi: 10.1186/s12881-019-0915-7 (PMC6849225; doi:10.1186/s12881-019-0915-7)
Supplement: Supplementary file 5 — Additional file 5: Table S2. Variants in the coding regions of the EIF2AK1–4 genes in Individulas AII1 and AII.2 (excluding synonymous variants). [file 12881_2019_915_MOESM5_ESM.pdf]

<u>Supplementary Table 1:</u> Variants in the coding regions of the *EIF2AK1-4* genes in Individulas AII1 and AII.2 (excluding synonymous variants)

SAMPLE ID	#CHROM	POS	REF	ALT	Zygosity	Effect	Putative_Impact	Gene_Name	Feature_ID	Transcript_BioType	HGVS.p	dbSNP142_ID	1000Gp3_AF	ESP6500_MAF_ALL
AII.2	chr15	40,246,148	TAAAAG	T	HOM	frameshift_variant	HIGH	EIF2AK4	NM_001013703.3	Coding	p.Lys187fs	.	.	.
AII.1	chr15	40,246,148	TAAAAG	T	HOM	frameshift_variant	HIGH	EIF2AK4	NM_001013703.3	Coding	p.Lys187fs	.	.	.
AII.2	chr15	40,265,799	A	G	HOM	missense_variant	MODERATE	EIF2AK4	NM_001013703.3	Coding	p.Glu556Gly	rs2307105	0.844449	A:0.057330
AII.1	chr15	40,265,799	A	G	HOM	missense_variant	MODERATE	EIF2AK4	NM_001013703.3	Coding	p.Glu556Gly	rs2307105	0.844449	A:0.057330
AII.2	chr15	40,269,052	G	A	HOM	splice_region_variant&intron_variant	LOW	EIF2AK4	NM_001013703.3	Coding	.	rs34298786	0.287141	A:0.336137
AII.1	chr15	40,269,052	G	A	HOM	splice_region_variant&intron_variant	LOW	EIF2AK4	NM_001013703.3	Coding	.	rs34298786	0.287141	A:0.336137
AII.2	chr15	40,322,552	G	T	HOM	splice_region_variant&intron_variant	LOW	EIF2AK4	NM_001013703.3	Coding	.	rs2250402	0.905551	G:0.069694
AII.1	chr15	40,322,552	G	T	HOM	splice_region_variant&intron_variant	LOW	EIF2AK4	NM_001013703.3	Coding	.	rs2250402	0.905551	G:0.069694
AII.2	chr7	6,080,853	GA	G	HET	splice_region_variant&intron_variant	LOW	EIF2AK1	NM_014413.3	Coding	.	rs34743814	.	.
AII.1	chr7	6,080,853	GA	G	HOM	splice_region_variant&intron_variant	LOW	EIF2AK1	NM_014413.3	Coding	.	rs34743814	.	.
AII.2	chr2	88,874,891	C	A	HET	missense_variant	MODERATE	EIF2AK3	NM_004836.5	Coding	p.Ala704Ser	rs1805165	0.720647	C:0.198293
AII.1	chr2	88,874,891	C	A	HET	missense_variant	MODERATE	EIF2AK3	NM_004836.5	Coding	p.Ala704Ser	rs1805165	0.720647	C:0.198293
AII.2	chr2	88,895,123	T	C	HET	missense_variant	MODERATE	EIF2AK3	NM_004836.5	Coding	p.Gln166Arg	rs13045	0.648962	T:0.265262
AII.1	chr2	88,895,123	T	C	HET	missense_variant	MODERATE	EIF2AK3	NM_004836.5	Coding	p.Gln166Arg	rs13045	0.648962	T:0.265262
AII.2	chr2	88,913,273	G	C	HET	missense_variant	MODERATE	EIF2AK3	NM_004836.5	Coding	p.Ser136Cys	rs867529	0.280551	C:0.198370
AII.1	chr2	88,913,273	G	C	HET	missense_variant	MODERATE	EIF2AK3	NM_004836.5	Coding	p.Ser136Cys	rs867529	0.280551	C:0.198370
AII.2	chr2	88,926,729	CCAG	C	HOM	inframe_deletion	MODERATE	EIF2AK3	NM_004836.5	Coding	p.Leu21del	rs144052332	0.796526	.
AII.1	chr2	88,926,729	CCAG	C	HOM	inframe_deletion	MODERATE	EIF2AK3	NM_004836.5	Coding	p.Leu21del	rs144052332	0.796526	.
