# Supplementary material for: Novel EIF2AK4 mutations in histologically proven pulmonary capillary hemangiomatosis and hereditary pulmonary arterial hypertension
Source: BMC Med Genet. 2019 Nov 11;20:176. doi: 10.1186/s12881-019-0915-7 (PMC6849225; doi:10.1186/s12881-019-0915-7)
Supplement: Supplementary file 6 — Additional file 6: Table S3. Variants in the coding regions of the EIF2AK4 gene network in Individulas AII1 and AII.2 (excluding synonymous variants). [file 12881_2019_915_MOESM6_ESM.pdf]

Supplementary Table 2: Variants in the coding regions of the *EIF2AK4* gene network in Individulas AII1 and AII.2 (excluding synonymous variants)

SAMPLE ID	#CHROM	POS	Zygosity	FILTER	Effect	Putative_Impact	Gene_Name	Feature_ID	Transcript_BioType	HGVS.p	dbSNP142_ID	1000Gp3_AF	ESP6500_MAF_ALL
AII.2	chr12	120,575,549	HOM	PASS	missense_variant	MODERATE	GCN1L1	NM_006836.1	Coding	p.Tyr2155Asp	rs3864938	0.999401	A:0.002197
AII.2	chr12	120,600,770	HET	PASS	missense_variant	MODERATE	GCN1L1	NM_006836.1	Coding	p.Val682Met	.	.	.
AII.2	chr22	39,917,515	HET	PASS	missense_variant	MODERATE	ATF4	NM_001675.4	Coding	p.Gln22Pro	rs4894	0.273762	C:0.302245
AII.2	chr17	59,544,863	HOM	PASS	splice_region_variant&intron_variant	LOW	TBX4	NM_018488.2	Coding	.	rs758596	0.347843	A:0.314701
AII.2	chr3	150,280,445	HOM	PASS	missense_variant&splice_region_variant	MODERATE	EIF2A	NM_032025.3	Coding	p.Thr97Ser	rs1132979	0.354233	G:0.292945
AII.2	chr3	150,281,340	HET	PASS	missense_variant	MODERATE	EIF2A	NM_032025.3	Coding	p.Leu111Phe	rs181238642	0.000599042	T:0.000431
AII.1	chr3	150,280,445	HOM	PASS	missense_variant&splice_region_variant	MODERATE	EIF2A	NM_032025.3	Coding	p.Thr97Ser	rs1132979	0.354233	G:0.292945
AII.1	chr3	150,281,340	HET	PASS	missense_variant	MODERATE	EIF2A	NM_032025.3	Coding	p.Leu111Phe	rs181238642	0.000599042	T:0.000431
AII.1	chr17	59,544,863	HET	PASS	splice_region_variant&intron_variant	LOW	TBX4	NM_018488.2	Coding	.	rs758596	0.347843	A:0.314701
AII.1	chr12	120,575,549	HOM	PASS	missense_variant	MODERATE	GCN1L1	NM_006836.1	Coding	p.Tyr2155Asp	rs3864938	0.999401	A:0.002197
